# Children under 15 kg with food allergy may be at risk of having epinephrine auto-injectors administered into bone

**DOI:** 10.1186/1710-1492-10-40

**Published:** 2014-08-01

**Authors:** Laura Kim, Immaculate FP Nevis, Gina Tsai, Arunmozhi Dominic, Ryan Potts, Jack Chiu, Harold L Kim

**Affiliations:** 1 Department of Anatomy and Cell Biology, McGill University, Montreal, Quebec, Canada; 2 Michael D. DeGroote School of Medicine, McMaster University, Hamilton, Ontario, Canada; 3 Schulich School of Medicine & Dentistry, Western University, London, Ontario, Canada; 4 Department of Biology, University of Waterloo, Waterloo, Ontario, Canada; 5 525 Belmont Avenue West, Suite 205, Kitchener N2M 5E2, Ontario, Canada

**Keywords:** Food allergy, Anaphylaxis, Skin-to-bone depth, Epinephrine, Auto-injector, Pediatric, Needle length

## Abstract

**Background:**

The Epipen® Jr and Allerject® 0.15 mg are currently the most commonly prescribed epinephrine auto-injectors (EAIs) for the management of anaphylaxis in pediatric patients in North America and Canada. To ensure rapid absorption, it should be administered intramuscularly into the anterolateral aspect of the thigh. We examined whether the 12.7-mm needle length of the Epipen® Jr and Allerject® 0.15 mg is adequate for delivering epinephrine intramuscularly in pediatric patients who weighed <15 kg.

**Methods:**

Consecutive pediatric patients with food allergy weighing <15 kg who required an EAI were included. Ultrasounds of the mid-anterolateral thigh were performed under minimal (min) and maximal (max) pressure. Skin-to-muscle depth (STMD) and skin-to-bone depth (STBD) measurements were completed. Baseline characteristics were compared between patients with a STBD_max_ <12.7 mm vs. ≥12.7 mm. Linear regression including variables such as age, sex, body mass index (BMI) and race was performed. The proportion of patients with a STBD_max_ <12.7 mm was compared in those weighing <10 kg vs. 10–14.9 kg.

**Results:**

One hundred patients were included; 29 (29%) had STBD_max_ <12.7 mm. Height (p = 0.02) and weight (p = 0.0002) differed significantly between the two groups. Approximately 19% of those weighing 10–14.9 kg and 60% of those <10 kg had a STBD_max_ <12.7 mm. In the multivariable regression analysis, BMI was found to be a significant predictor of STBD_max_.

**Conclusions:**

A large proportion of children <15 kg prescribed an EAI is at risk of having the auto-injector administered into bone. Since alternative EAIs with shorter needle lengths are not currently available, EAIs should be prescribed with appropriate counselling in this population.

## Background

Anaphylaxis has been identified as an important cause of morbidity and mortality [1]. Although epidemiological data on anaphylaxis are limited, a study from Spain revealed an incidence of 103 episodes per 100,000 person years [2]. A significant number of hospital admissions are due to anaphylaxis and, although less common, death from anaphylaxis can also occur. A recent analysis of fatalities in Brazil suggests that the accuracy of diagnostic codes using International Classification of Diseases-10 (ICD-10) may miss a significant number of fatal anaphylaxis cases [3,4]. A major risk factor for death from anaphylaxis is the delayed use or failure to use epinephrine [5]. One study found that in infants with anaphylaxis, only 30% received epinephrine injections [6].

Currently, it is recommended that epinephrine be administered intramuscularly (to allow for rapid absorption) since subcutaneous delivery has been shown to result in slower absorption [7,8]. For the outpatient management of anaphylaxis, EAIs are generally recommended. The Epipen® Jr and Allerject® 0.15 mg, for example, are widely prescribed for pediatric patients with anaphylaxis. These EAIs have a needle length of 12.7 mm and are indicated for at-risk patients weighing between 15 and 30 kg [9,10]. In clinical practice, however, these EAIs are often used in children <15 kg. There are also some published medical statements suggesting that they can be prescribed in children weighing 10–25 kg [11]. In children weighing <10 kg, there are no formal guidelines or recommendations supporting the use of any commercially available EAI; nonetheless, the EAIs are often prescribed in this patient population as well.

Given the increased rate of obesity in children, there have been concerns that the EAI needles may not be long enough for intramuscular delivery in the pediatric population. Results of a study performed in children not at risk of anaphylaxis who presented to the radiology or emergency departments of a tertiary-care hospital in Phoenix, Arizona suggested that the needle of the EAIs might be too short to reach the intramuscular space in a significant number of children [12]. However, ultrasound measurements without pressure application were used in this study. This is a noteworthy limitation since EAIs require pressure to inject the needle.

In the present study, we sought to evaluate whether children weighing <15 kg who are at risk of anaphylaxis would appropriately receive the EAI into the intramuscular compartment. Of note, the Epipen® Jr and Allerject® 0.15 mg are officially indicated for children between 15 and 30 kg. But it is often prescribed in children <15 kg because there is no clinically accepted alternative with a lower dose of epinephrine. Originally, we postulated that, due to obesity, a significant number of these children would receive the injections subcutaneously including those <15 kg. However, with applied pressure, we identified that some children may receive injections into the bone since their skin-to-bone depth at maximal pressure (STBD_max_) is less than the needle length of the EAIs (12.7 mm). Therefore, we prospectively measured the likelihood of children <15 kg at risk of anaphylaxis having a STBD_max_ less than 12.7 mm.

## Methods

All of the patients’ parents or guardians provided written, informed consent prior to participating in this study. The Lawson Health Research Institute Research Ethics Board at Western University in London, Ontario, Canada approved the study.

**Figure 1:**
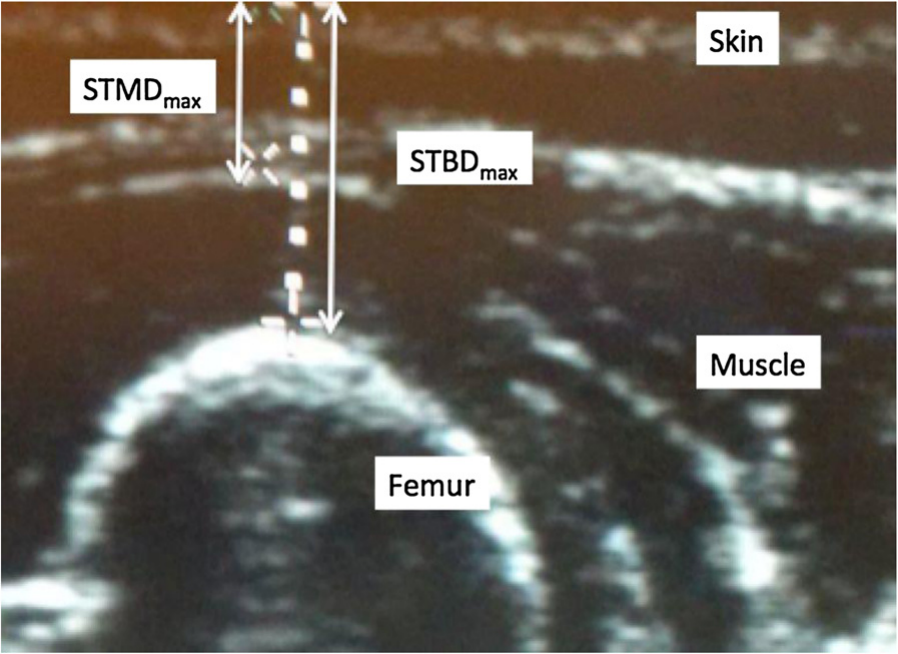
**Ultrasound image of anterolateral thigh.** STMD_max_: skin-to-muscle depth with maximal pressure; STBD_max_: skin-to-bone depth with maximal pressure.

Consecutive pediatric patients with confirmed food allergy weighing less than 15 kg who would benefit from EAI prescriptions in an allergist’s office were included in this trial. The subjects were assessed from July 2012 to November 2013. All subjects’ parents/guardians agreed to participate in the study. An ultrasound on the anterolateral aspect of the right mid thigh (the recommended site for injections with EAIs) was performed to measure four distances of tissue depths: skin-to-muscle depth with minimal pressure (STMD_min_), skin-to-muscle depth with maximal pressure (STMD_max_), skin-to-bone depth with minimal pressure (STBD_min_) and skin-to-bone depth with maximal pressure (STBD_max_) (see Figure 1). The investigator applied the pressure while performing the ultrasound measurements on each subject. The estimated maximal force was 2–8 lbs. All ultrasounds were completed by a single physician using a Sonosite Titan® ultrasound machine.

The primary outcome variable was the proportion of subjects with a STBD_max_ less than 12.7 mm. These subjects may be at risk of injecting epinephrine completely through the muscle and into the femur. Baseline characteristics between patients with a STBD_max_ <12.7 mm and those with a STBD_max_ ≥12.7 mm were analyzed and compared using the Student’s t-test/Mann Whitney U test for continuous variables and Chi-square/Fischer’s exact test for categorical variables. Linear regression analysis was performed including variables such as age, sex, race, and body mass index (BMI). The likelihood of the STBD_max_ being <12.7 mm was also calculated for the patient cohorts weighing <10 kg and 10–14.9 kg.

## Results

**Figure 2:**
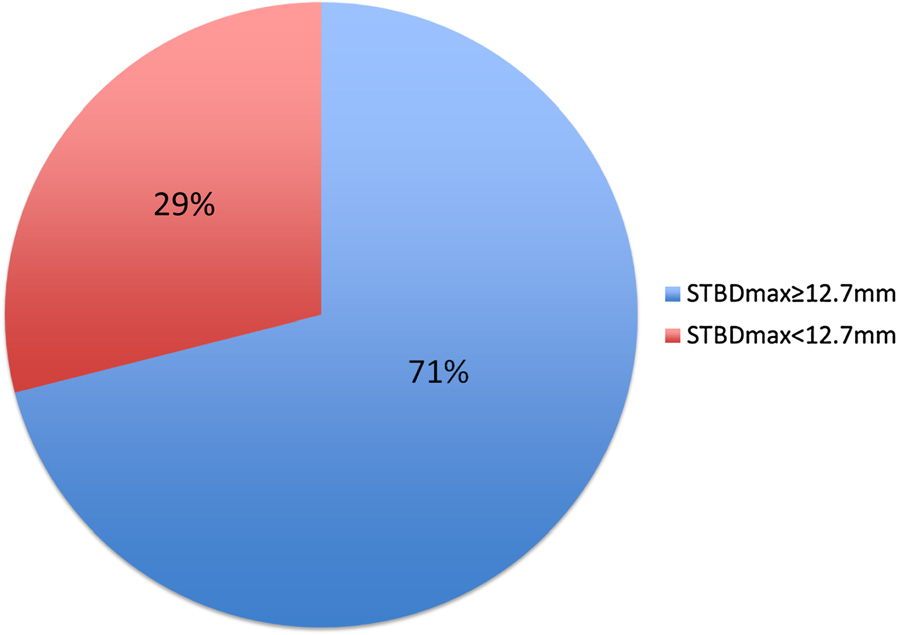
**Proportion of subjects with STBD**_**max **_**<12.7 mm or ≥12.7 mm.**

A total of 100 participants weighing <15 kg were included in this study; 29 subjects (29%) had a STBD_max_ <12.7 mm (see Figure 2). Baseline characteristics of patients with a STBD_max_ <12.7 mm vs. ≥12.7 mm are compared in Table 1. Weight (p = 0.0002) and height (p = 0.02) were significantly different between the two groups. Interestingly, mean BMI did not differ between the two comparison groups (p = 0.36). The mean STMD and STBD without pressure also differed significantly between the two groups (p < 0.05) (see Table 1). There were no patients with STMD_max_ >12.7 mm.

**Table 1:** Baseline characteristics of the patient cohort

**Characteristic**	**Total**	**Patients with STBD**_**max **_**≥12.7 mm**	**Patients with STBD**_**max **_**<12.7 mm**	**P value**
	**(n = 100)**	**(n = 71)**	**(n = 29)**	
Age (months), median (IQR)	17 (45)	18 (45)	16 (31)	0.19
Males, n (%)	55 (55)	39 (55)	16 (55)	0.98
White race, n (%)	78 (78)	57 (80)	21 (72)	0.38
Weight (kg), mean (SD)	11.5 (2.2)	12.1 (1.9)	10.2 (2.3)	0.0002
Height (m), mean (SD)	0.84 (0.1)	0.85 (0.1)	0.79 (0.1)	0.02
BMI (kg/m^2^), mean (SD)	16.4 (1.9)	16.5 (1.9)	16.1 (1.3)	0.36
STMD_max_ (mm), mean (SD)	6.4 (1.4)	6.7 (1.4)	5.5 (0.9)	0.0003
STMD_min_ (mm), mean (SD)	7.8 (1.8)	8.1 (1.8)	7.1 (1.6)	0.02
STBD_min_(mm), mean (SD)	25.1 (4.3)	26.2 (3.8)	22.3 (4.0)	0.0001

IQR: interquartile range; SD: standard deviation; BMI: body mass index; STMD_min_: skin-to-muscle depth with minimal pressure; STMD_max_: skin-to-muscle depth with maximal pressure; STBD_min_: skin-to-bone depth with minimal pressure; STBD_max_: skin-to-bone depth with maximal pressure.

**Figure 3:**
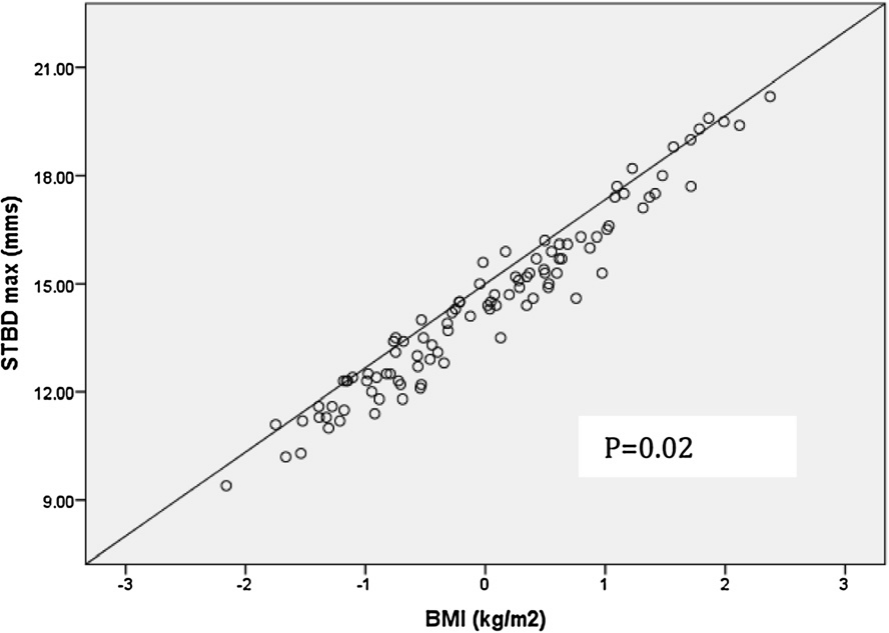
**Regression line showing the association between BMI and STBD**_**max**_**.** BMI: body mass index; STBD_max_: skin-to-bone depth with maximal pressure.

**Figure 4:**
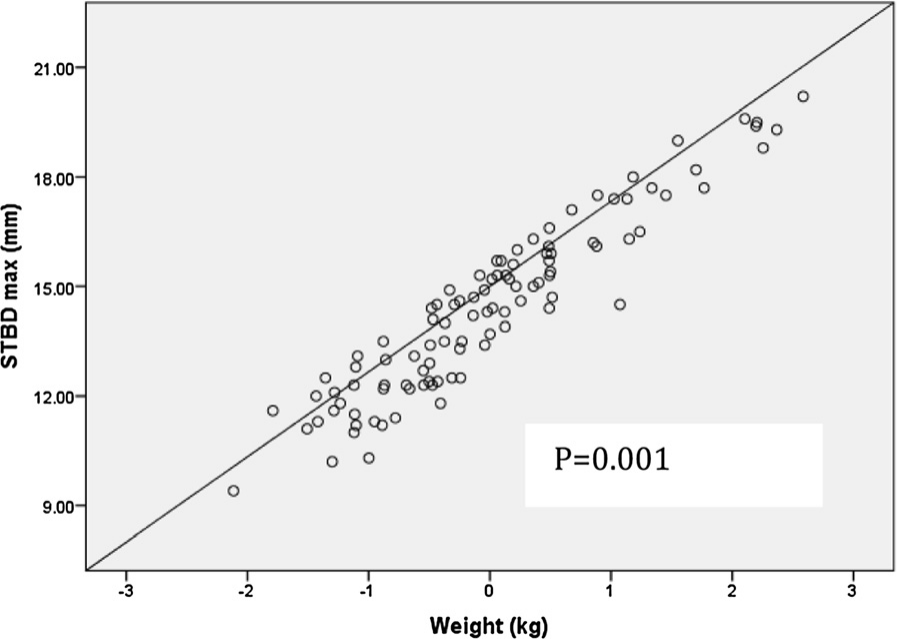
**Regression line showing the association between weight and STBD**_**max**_**.** STBD_max_: skin-to-bone depth with maximal pressure.

Multivariable linear regression analysis showed BMI (p = 0.02; Figure 3) to be significantly associated with STBD_max_ pressure following adjustment for age, sex and race. Since mean weight of the participants was significantly different between the two groups, multivariable linear regression analysis was repeated using age, sex, race and weight as independent variables. Weight (kg) was found to be the strongest predictor of STBD_max_ (p = 0.001; Figure 4).

**Figure 5:**
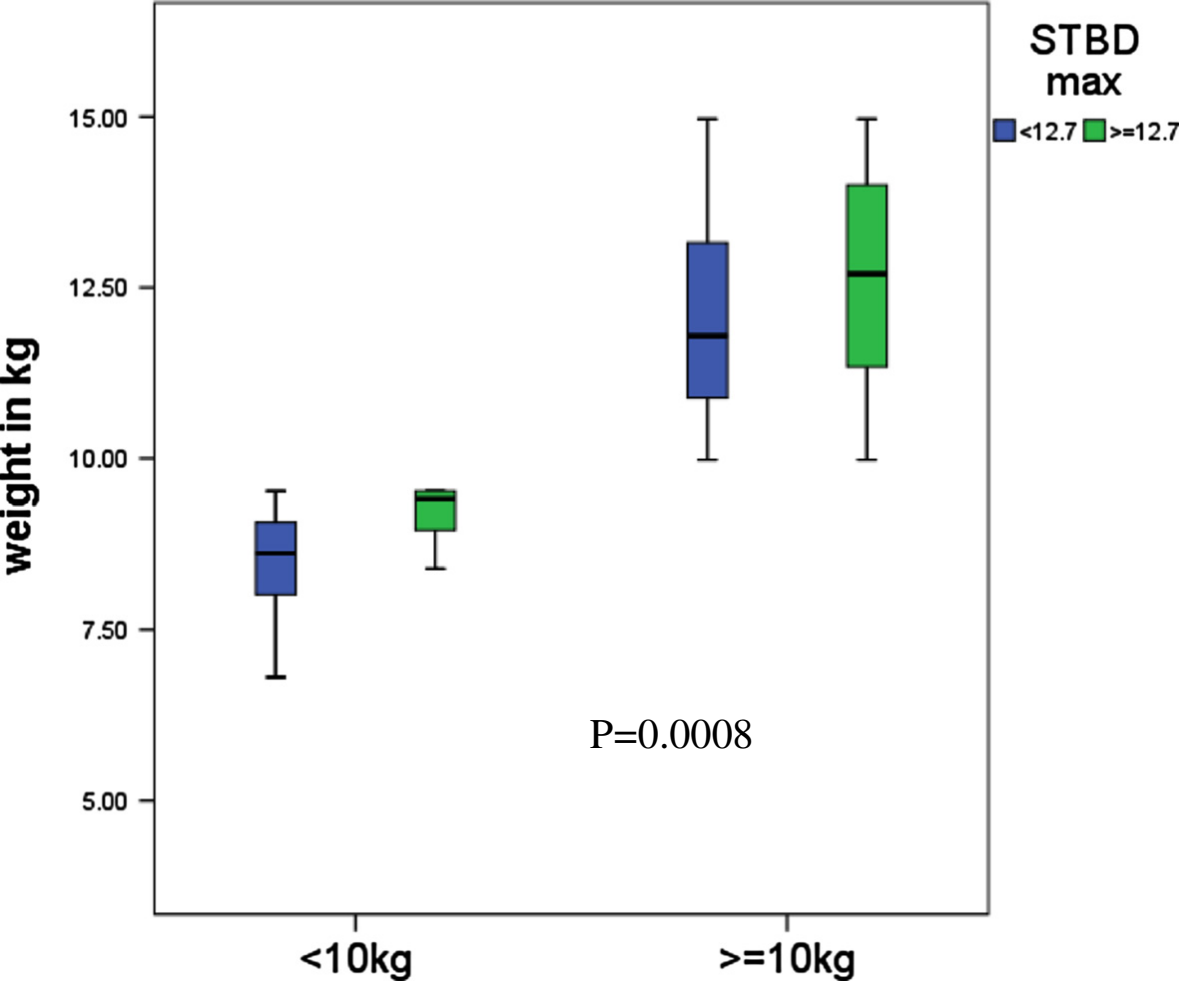
Box plots of different weight groups among the two comparison groups.

Table 2 and Figure 5 show the proportion of patients with a STBD_max_ <12.7 mm or ≥12.7 mm according to different weight groupings. Approximately 19% of patients between 10 and 14.9 kg had a STBD_max_ <12.7 mm compared with 60% of those weighing under 10 kg (p = 0.0008).

**Table 2:** **Proportion of subjects with STBD**_**max **_**less than or ≥12.7 mm according to weight**

**Weight (kg)**	**No. of patients with STBD**_**max **_**≥12.7 mm**	**Number of patients with STBD**_**max **_**<12.7 mm**	**P value**	**P value**
	**n (%)**	**n (%)**		
<10	10 (40)	15 (60)	0.0002	0.0008
10 to 14.9	61 (81)	14 (19)	0.0002	

STBD_max_: skin-to-bone depth with maximal pressure.

## Discussion

This prospective study examined whether the length of the EAI needles are adequate for delivering epinephrine intramuscularly in pediatric patients at risk of anaphylaxis who weighed <15 kg. Importantly, although the Epipen® Jr and the Allerject® 0.15 mg are officially indicated for children between 15 and 30 kg in Canada, they are often prescribed in children <15 kg as there is no clinically available EAI that delivers a lower dose of epinephrine. Ultrasound measurements of the mid anterolateral thigh were performed with pressure application to simulate the pressure required to inject an auto-injector. Although we originally believed that a significant proportion of these children was at risk of receiving the auto-injector subcutaneously (rather than intramuscularly) due to obesity, we found that a significant proportion of these children were at risk of receiving the auto-injectors into the bone. Physicians should be aware of this potential risk.

Currently, there are no published clinical studies assessing whether the 12.7-mm length of the EAI needles are adequate for delivering epinephrine intramuscularly in pediatric patients weighing <15 kg at risk of anaphylaxis. All of our subjects had a STMD_max_ <12.7 mm, suggesting that none of these children were at risk of having the auto-injector administered into the subcutaneous space. Our findings contrast those reported by Stecher et al. [12] who identified 12% of children weighing <30 kg that were at risk of receiving the EAI into the subcutaneous space. Age and BMI correlated with STMD in these children. It is important to note that this study enrolled children (1–12 years of age) presenting to the radiology or emergency departments of a tertiary-care hospital in a non-consecutive fashion. Subjects were not at risk of anaphylaxis, and ultrasounds with pressure and measurements to the femur were not completed. Almost half of the study population was Hispanic and children who weighed <15 kg were not analyzed separately. Our study, on the other hand, assessed primarily Caucasian children <15 kg who were at risk of anaphylaxis. Also, ultrasounds with pressure were performed to simulate how auto-injectors are given in the "real-life" setting.

Our study found that almost 30% of children < 15 kg had a STBD_max_ <12.7 mm and were therefore at risk of receiving epinephrine into the bone. Patients weighing <10 kg were at even greater risk since 60% of these subjects had a STBD_max_ <12.7 mm. We believe these findings are clinically important, particularly since there are currently no randomized controlled trials evaluating or comparing subcutaneous, intramuscular, periosteal, cortical, intraosseous or intravenous epinephrine administration in patients with anaphylaxis. However, the ethical considerations in completing studies of this nature may be prohibitive. In children with a history of anaphylaxis, epinephrine injected intramuscularly compared to subcutaneously has been shown to lead to higher serum epinephrine levels more rapidly [13,14]. This more rapid peak in epinephrine levels has been the basis for recommending intramuscular epinephrine administration as the standard of care for anaphylaxis. Intravenous or intraosseous epinephrine is reserved for severe, life-threatening anaphylaxis with associated hypotension, airway swelling, severe bronchospasm or inadequate response to intramuscular epinephrine. Intravenous epinephrine should be given at a 1/100,000 strength at a maximum infusion rate of 10 mcg/min [1,11,15]. The Epipen® Jr and Allerject® 0.15 mg provide a total dose of epinephrine of 0.15 mg at 1/2,000 and 1/1,000 strengths respectively [9,10]. These strengths are 50 and 100 times the concentration suggested for intraosseous infusion respectively. Importantly, there are no studies to confirm that the auto-injectors would penetrate through the femur of children. But we believe the auto-injector needle would penetrate the bone as supported by a case report of an adult female experiencing an accidental injection that went completely through the bone of a distal phalanx [16]. Also, the thickness of the cortical bone of the femur has not been formally studied in young children. Animal studies confirm that intraosseous epinephrine administration leads to similar serum epinephrine levels as intravenous administration [17,18]. Although the intravenous or intraosseous route of administration should be used in the appropriate clinical setting, there have been reports of severe side effects with intravenous epinephrine administration. For example, a 29-year-old woman had a myocardial infarction after receiving 0.1 mL of 1/10,000 intravenous epinephrine [19]. Sullivan reported two patients who had ventricular tachycardia after receiving a 5-mL intravenous injection of 1/10,000 epinephrine [20]. There is also a report of a 5-month-old child weighing 7 kg who presented with an allergic reaction to an emergency department and received 0.7 mL of 1/1,000 subcutaneous epinephrine twice and then 0.7 mL of 1/1,000 epinephrine intravenously [21]. The infant had a cardiac arrest and could not be resuscitated. Although these reports involve cases where epinephrine was given at higher doses or concentrations than currently recommended, they illustrate the potential risks of intravenous and intraosseous epinephrine.

Children weighing <15 kg with a STBD_max_ <12.7 mm who are prescribed an EAI are at risk of injecting a more concentrated and higher-than-recommended dose of epinephrine into the intraosseous space. There are various strategies that might be considered to help the clinician to deal with this issue. Ideally, all children requiring an EAI should have a STBD_max_ measurement with ultrasound to identify those who may be at risk of intraosseous administration. In at-risk children, other forms of injectable epinephrine could be considered, such as the provision of separate syringes (with variable needle lengths) and vials of epinephrine. However, one study suggests that the parents of these children may not be able to draw up the proper doses of epinephrine reliably in a reasonable timeframe to manage anaphylaxis [13]. Another strategy would be to instruct the child’s parent or care provider to squeeze the leg and muscle at the site of injection so that the EAI does not compress the muscle. In most patients, we believe this would lead to intramuscular injection. If using this strategy, persons injecting must be cautious not to inject the device into their own hand. Manufacturers should also consider developing auto-injectors with variable needle lengths (and doses) and/or devices that require less pressure for administration, as this would increase the likelihood of intramuscular injection. More thorough studies assessing the pharmacokinetics and pharmacodynamics of injecting epinephrine into the periosteum, cortical bone or intraosseous space are also required.

The main strengths of this study were that the patient cohort included children at risk of anaphylaxis who weighed <15 kg, the STBD_max_ was used as the primary variable, and ultrasound measurements were taken in the proper location for EAI application. The findings of this research reveal a potential shortcoming in our current approach to anaphylaxis.

One limitation of this study was that it was performed in only one clinic. It is possible that the findings may differ if utilizing a multicentre study design. Therefore, we suggest that a similar study be replicated in other centres. A second limitation was that one physician performed all of the ultrasound measurements in an unblinded fashion. Nonetheless, we feel the data collected were accurate since measurements were simple to perform and easily reproducible. The ultrasound machine included an easy-to-use tool to accurately determine the measurements for each variable assessed in our study. A third limitation is that the physician applied the maximum pressure to the thigh without using any method of formally quantifying the pressure applied with the ultrasound probe. We believe that, in many children, "real-life" use of the auto-injector may actually lead to more muscle compression and/or an increased risk of injecting into the bone than was noted in our study. This may occur because greater force may be applied by parents injecting the auto-injectors, the surface area of the currently available auto-injectors are less than that of the ultrasound probe and/or the device may be given in an area were muscle thickness is less than that in the mid anterolateral thigh. In future studies, we suggest that the pressures required to trigger the various types of auto-injectors be measured and that these pressures be applied for ultrasound measurements. The pressure required and the depth of muscle compression may vary for each device and, possibly, for each individual patient. A final limitation of this study is that the data were not analyzed to address other EAIs aside from the Epipen® Jr and the Allerject® 0.15 mg. The risk is likely similar for injection into the bone with the Epipen® Jr and the Allerject® 0.15 mg. But the surface area and pressure required to inject these devices may affect the depth of injection. These products as well as products that become available later should be compared in future studies.

## Conclusions

A significant proportion of children weighing under 15 kg who require a prescription for an EAI is at risk of receiving epinephrine injections into the bone. Given this risk, EAIs should be prescribed with caution in this patient population. At this time, there are no alternative EAIs with needle lengths shorter than 12.7 mm. We recommend further research in this area using larger patient cohorts and multicentre study designs.

## Abbreviations

EIA: Epinephrine auto-injector; STMD: Skin-to-muscle depth; STMD_min_: Skin-to-muscle depth with minimal pressure; STMD_max_: Skin-to-muscle depth with maximal pressure; STBD: Skin-to-bone depth; STBD_min_: Skin-to-bone depth with minimal pressure; STBD_max_: Skin-to-bone depth with maximal pressure; BMI: Body mass index.

## Competing interests

HK has been on an advisory board for Sanofi Canada and the speakers’ bureau for Pfizer Canada. GT, LK, IN, RP, JC, AD declare that they have no competing interests.

## Authors’ contributions

LK, GT, RP, JC, and HK were responsible for the conception and design of the study. LK and RP were responsible for the acquisition of the data. IN and AD were responsible for data analysis. LK and IN drafted the manuscript. All of the authors contributed substantially to the interpretation of the data, critically revised the manuscript for important intellectual content, approved the final version submitted for publication and agree to act as guarantors of the work.
